# Tobacco Smoke Exposure and Oxidative Stress: The Role of Circulating Lipopolysaccharides in Heated and Conventional Products

**DOI:** 10.3390/antiox14111316

**Published:** 2025-10-31

**Authors:** Lorenzo Loffredo, Enrico Maggio, Simona Bartimoccia, Arianna Magna, Chiara Maria Totè, Chiara Bagnato, Bianca Laura Cinicola, Federica Armeli, Angela Leonardo, Alessandra D’Amico, Ernesto Greco, Giacomo Frati, Giuseppe Biondi-Zoccai, Alberto Spalice, Antonio Angeloni, Pasquale Pignatelli, Francesco Violi, Anna Maria Zicari, Roberto Carnevale, Smoking Prevention Study Group

**Affiliations:** 1 Department of Medical and Cardiovascular Sciences, Sapienza University of Rome, 00185 Rome, Italy; enrico.maggio@uniroma1.it (E.M.); simona.bartimoccia@uniroma1.it (S.B.); arianna.magna@uniroma1.it (A.M.); chiaramaria.tote@uniroma1.it (C.M.T.); chiara.bagnato@uniroma1.it (C.B.); pasquale.pignatelli@uniroma1.it (P.P.); 2 Interdisciplinary Department of Well-Being, Health and Environmental Sustainability (BESSA), Sapienza University of Rome, 02100 Rieti, Italy; antonio.angeloni@uniroma1.it; 3 Department of Maternal Infantile and Urological Sciences, Sapienza University of Rome, 00161 Rome, Italy; bianca.cinicola@uniroma1.it (B.L.C.); alberto.spalice@uniroma1.it (A.S.); annamaria.zicari@uniroma1.it (A.M.Z.); 4 Department of Medical-Surgical Sciences and Biotechnologies, Sapienza University of Rome, 04100 Latina, Italy; federica.armeli@uniroma1.it (F.A.); ang.leonardo.28@gmail.com (A.L.); giacomo.frati@uniroma1.it (G.F.); giuseppe.biondizoccai@uniroma1.it (G.B.-Z.); roberto.carnevale@uniroma1.it (R.C.); 5 Department of Health and Life Sciences, European University of Rome, 00100 Rome, Italy; alessandra.damico@uniroma1.it (A.D.); ernesto.greco@unier.it (E.G.); 6 IRCCS Neuromed, 86077 Pozzilli, Italy; 7 Maria Cecilia Hospital, GVM Care & Research, 48033 Cotignola, Italy; 8 Sapienza University of Rome, 00185 Rome, Italy; francesco.violi@uniroma1.it

**Keywords:** smoking, heated tobacco products, cigarettes, LPS, lipopolysaccharides, endothelial dysfunction, flow-mediated dilation, NOX-2, NADPH-oxidase 2, atherosclerosis

## Abstract

**Background:** Exposure to tobacco smoke, from conventional tobacco cigarettes (CTC) or heated tobacco products (HTPs), increases oxidative stress, causing endothelial dysfunction and higher cardiovascular risk. It is unclear whether smoke exposure also promotes low-grade endotoxemia, potentially activating NADPH oxidase and further impairing endothelial function. This study assessed serum lipopolysaccharide (LPS) levels in children and adults actively or passively exposed to conventional cigarette smoke or HTPs, compared with non-exposed controls. **Methods:** We conducted a cross-sectional study comprising 26 children passively exposed to HTPs, 26 children exposed to CTC, and 26 unexposed controls, as well as 20 adult chronic HTP users, 20 chronic CTC, and 20 non-smoking adults. Circulating LPS was measured alongside oxidative stress markers (NOX2, H_2_O_2_), endothelial function, intestinal permeability (zonulin), and nicotine exposure (serum cotinine). **Results:** Exposed children had higher cotinine, LPS, and zonulin than controls, with no differences between HTP and CTC groups. Multiple linear regression analysis identified cotinine (β = 0.343; *p* = 0.005) and zonulin (β = 0.441; *p* < 0.001) as independent LPS predictors. In adults, LPS and zonulin were higher in both smoker groups versus controls; zonulin (β = 0.477; *p* < 0.001) and nitric oxide bioavailability (β = −0.307; *p* = 0.007) independently predicted LPS. **Conclusions:** Passive and active exposure to CTC or HTPs increases low-grade endotoxemia and zonulin, potentially driving NOX2-mediated oxidative stress.

## 1. Introduction

Tobacco consumption remains one of the leading preventable causes of morbidity and mortality worldwide, primarily through its contribution to cardiovascular complications and cancer [1,2]. Traditional combustible cigarettes release thousands of toxic compounds, including reactive aldehydes, heavy metals, and particulate matter, which contribute to systemic inflammation and the development of chronic diseases [1]. In recent years, heated tobacco products (HTPs) have been marketed as reduced-risk alternatives to conventional smoking, based on the rationale that aerosol generation at lower temperatures may decrease exposure to harmful and potentially harmful constituents [1,3]. However, accumulating evidence suggests that both cigarette smoke and HTPs aerosols may still exert detrimental effects on human health [1,4].

A key mechanism linking tobacco exposure to systemic disease is low-grade endotoxemia, characterized by chronically elevated plasma levels of lipopolysaccharide (LPS), a structural component of Gram-negative bacteria [5]. Smoking-induced gut dysbiosis and impaired intestinal barrier integrity facilitate LPS translocation into the circulation [6], which is further aggravated by the direct presence of endotoxins adsorbed onto smoke particles [7]. Even low-grade increases in circulating LPS can activate Toll-like receptor 4 (TLR4) signaling, initiating immune responses that amplify systemic inflammation [5].

Among the downstream mediators, NADPH oxidase 2 (NOX2) plays a pivotal role in promoting systemic oxidative stress [8,9]. LPS-TLR4 engagement activates NOX2 in innate immune cells, leading to enhanced production of reactive oxygen species (ROS) [5]. Excessive ROS contributes to oxidative stress, endothelial dysfunction, and vascular injury, thereby creating a mechanistic link between low-grade endotoxemia and the cardiovascular complications observed in smokers [5,10]. Increased circulating levels of NOX2-derived markers have been described not only in active smokers but also in individuals passively exposed to second- and third-hand smoke [11,12,13].

Children exposed to secondhand smoke represent a particularly vulnerable population. Passive exposure during critical windows of immune and metabolic development may predispose to long-term inflammatory phenotypes and NOX2-driven oxidative damage, thereby amplifying the burden of chronic disease in adulthood [12,13]. Meanwhile, chronic smokers, whether using conventional cigarettes or HTPs, may exhibit distinct profiles of endotoxemia, NOX2 activation [11], and endothelial dysfunction, although comparative human data remain scarce.

In this context, studying the impact of traditional and heated tobacco smoking on low-grade endotoxemia, with a focus on LPS-induced NOX2 activation, in both passive pediatric exposure and chronic smokers is crucial.

The aim of this study was to evaluate serum LPS levels in children and adults who were actively or passively exposed to conventional cigarette smoke or HTPs, compared with non-exposed controls, and to investigate the correlation between LPS levels, oxidative stress, and endothelial function.

## 2. Materials and Methods

The populations of children exposed to passive smoking by HTPs and traditional tobacco and chronic smokers of HTPs or traditional tobacco were taken from 2 studies previously published by the same authors [11,12].

### 2.1. Aim of the Study

This study was designed as an observational cross-sectional investigation.

The primary aim was to assess circulating LPS levels in first-hand smokers (active smokers) and second-hand smokers (individuals exposed to passive smoke) of heated tobacco products (IQOS, Philip Morris, New York, NY, USA) and traditional combustible tobacco.

A secondary objective was to investigate the relationship between circulating LPS and several biomarkers, including soluble NOX2-derived peptide (sNOX2-dp), serum isoprostanes, hydrogen peroxide (H_2_O_2_) concentrations, and hydrogen peroxide breakdown activity (HBA, a measure of antioxidant capacity). Associations were also explored with markers of endothelial dysfunction—namely nitric oxide (NO) bioavailability and flow-mediated dilation (FMD)—as well as with platelet activation, assessed by serum levels of soluble CD40 ligand (sCD40L) and soluble P-selectin (sP-selectin).

Two populations, including both children and adults, were selected to evaluate the effects of active and passive exposure to traditional tobacco cigarettes or HTPs on endotoxemia levels, and to correlate these with markers of oxidative stress, endothelial dysfunction, and platelet activation.

### 2.2. Study Population: Passive Smoke Exposure and Controls

To investigate the effects of passive smoke exposure, 78 children were recruited: 26 exposed to passive smoke from HTPs (IQOS, Philip Morris, New York, NY, USA), 26 exposed to traditional combustible tobacco smoke, and 26 non-exposed children serving as controls.

Specifically, the eligibility criteria included children and adolescents aged 2 to 18 years, in apparent good health (defined as the absence of known chronic or acute diseases), who had been exposed or not exposed to passive smoking for at least six months. 

Exclusion criteria were: obesity or severe underweight, chronic inflammatory disease, diabetes mellitus, dyslipidemia, active or past smoking, cardiopulmonary disease, severe nephropathy, liver disease, neuromuscular disorders, or vitamin supplementation. Children with one parent smoking traditional tobacco and the other using HTPs were also excluded.

Written informed consent was obtained from parents in accordance with Italian regulations. The study protocol was approved by the Ethics Committee of Sapienza University of Rome (ref. no. 4929; protocol no 173/18, date of approval 22 February 2018) and conducted in accordance with the Declaration of Helsinki.

### 2.3. Study Population: Chronic Active Smokers and Controls

To investigate the effects of chronic active smoking (first-hand exposure), 60 healthy adult participants were recruited and blood donors who provided written informed consent.

Of these, 20 had smoked HTPs for at least one month, 20 had smoked tobacco combustible cigarettes for at least one month, and 20 were non-smokers serving as controls. All HTP users were former conventional cigarette smokers who had. switched exclusively to HTPs. Their mean duration of HTP use was 1.5 ± 0.5 years. To be classified as chronic smokers, participants had to use only HTPs for more than one month [11].

Participants had not taken vitamin E, antioxidant supplements, or antiplatelet drugs during the month preceding the study. Female participants were not menstruating at the time of blood collection. The study was approved by the Ethics Committee of Sapienza University of Rome (ref. no. 3241; protocol no. 813/14, date of approval 26 June 2014) and conducted in compliance with the Declaration of Helsinki.

### 2.4. Sample Collection

Fasting blood samples were collected between 8:00–9:00 a.m. from the antecubital vein into Vacutainer tubes (with or without anticoagulants). Plasma and serum aliquots were separated and stored at −80 °C until analysis.

#### 2.4.1. Biochemical and Functional Assessments

##### NO_2_^−^/NO_3_^−^ (Serum Nitric Oxide Metabolites)

Serum nitric oxide (NO) concentration was determined using a colorimetric assay based on the quantification of nitrite/nitrate (NO_2_^−^/NO_3_^−^) (Arbor Assays, Ann Arbor, MI, USA). Nitrate (NO_3_^−^) in the sample was reduced to nitrite (NO_2_^−^) by nitrate reductase, after which the total nitrite was detected by reaction with Griess reagents, leading to the formation of a colored azo compound with absorbance measured at 540 nm. Results were expressed in μM. Intra- and inter-assay coefficients of variation (CVs) were both <10%.

#### 2.4.2. 8-Isoprostane (8-Iso-PGF_2_α)

Serum concentrations of 8-iso-Prostaglandin F2α (8-iso-PGF2α) were measured using commercial competitive enzyme-linked immunosorbent assay (ELISA) kits (Abcam, Cambridge, UK; DRG International, Springfield, NJ, USA). Serum samples and standards were added to microplate wells pre-coated with anti–8-iso-PGF2α antibodies and incubated with an enzyme-conjugated tracer. After unbound components were removed by washing, a colorimetric substrate was applied. The enzymatic reaction generated a color change inversely proportional to the 8-iso-PGF2α concentration. Absorbance was read at 450 nm (Abcam) or 405 nm (DRG) using a microplate reader. Results were expressed in pg/mL. Intra- and inter-assay CVs were <10%.

#### 2.4.3. Soluble NOX2-Derived Peptide (sNOX2-dp)

The soluble NOX2-derived peptide (sNOX2-dp) was measured by ELISA. This peptide, released upon platelet activation, is recognized by a monoclonal antibody directed against amino acids 224–268 of the extracellular portion of NOX2, the catalytic subunit of NADPH oxidase. Enzymatic activity was quantified spectrophotometrically by the increase in absorbance at 450 nm. Results were expressed in pg/mL, with intra- and inter-assay CVs of 8.95% and 9.01%, respectively.

#### 2.4.4. Hydrogen Peroxide (H_2_O_2_)

Serum H_2_O_2_ concentration was determined using a colorimetric assay (Abcam, Cambridge, UK), according to the manufacturer’s instructions [14]. The final product was read at 450 nm, and results were expressed in μM. Intra- and inter-assay CVs were <10%.

#### 2.4.5. Hydrogen Peroxide Breakdown Activity (HBA)

Serum antioxidant activity was assessed using the HBA kit (Hydrogen Peroxide Breakdown Activity, Aurogene, code HPSA-50), which quantifies the ability of samples to degrade H_2_O_2_. Results were expressed as percentage HBA, calculated according to the kit formula.

#### 2.4.6. Soluble P-Selectin

Plasma soluble P-selectin concentration was measured using a commercial ELISA kit (DRG International, Springfield, NJ, USA). Results were expressed in ng/mL. Intra- and inter-assay CVs were 4.3% and 6.1%, respectively.

#### 2.4.7. Soluble CD40 Ligand (sCD40L)

Serum sCD40L levels were determined by solid-phase ELISA (DRG International, Springfield, NJ, USA). Results were expressed in ng/mL. Intra- and inter-assay CVs were 3.2% and 4.3%, respectively.

#### 2.4.8. Cotinine

Serum cotinine was measured using a human cotinine ELISA kit (Sigma Aldrich, St. Louis, MO, USA), based on a solid-phase competitive immunoassay. Samples and cotinine–enzyme conjugate were incubated in wells coated with anti-cotinine antibodies. After substrate addition, color intensity was inversely proportional to sample cotinine concentration. Absorbance was read at 550 nm, and results were expressed in ng/mL. Intra- and inter-assay CVs were <10%.

#### 2.4.9. Lipopolysaccharide (LPS)

Serum LPS concentrations were quantified using a commercial ELISA kit (Cusabio, Wuhan, China). Standards and samples were added to a microplate pre-coated with an anti-LPS antibody and incubated for 2 h at room temperature. Following incubation, absorbance was measured at 450 nm. Results were expressed as pg/mL, with both intra- and inter-assay coefficients of variation below 10%.

#### 2.4.10. Zonulin

Serum zonulin levels were measured using a commercial ELISA kit (Elabscience, Houston, TX, USA). A zonulin-specific antibody was pre-coated onto a microplate; 100 μL of standards and patient serum samples were added and incubated for 90 min at 37 °C. A biotinylated zonulin-detection antibody and horseradish peroxidase (HRP)–avidin conjugate were then added. Results were expressed in ng/mL. Intra- and inter-assay CVs were <10%.

### 2.5. Flow-Mediated Dilation (FMD)

Ultrasound evaluation of baseline brachial artery diameter and endothelium-dependent dilation (FMD) was performed according to published guidelines [15,16,17] and previous reports [9]. In brief, assessments were performed between 8:00 a.m. and 10:00 a.m. with participants resting in the supine position in a temperature-controlled room (22.8 °C). A 7.5-MHz linear array transducer ultrasound system (HS30, Samsung, Seoul, Republic of Korea), equipped with electronic calipers, was used to measure the brachial artery 3–7 cm above the antecubital crease. Dedicated vascular software provided two-dimensional imaging as well as color and spectral Doppler. A sphygmomanometer cuff was placed on the forearm and inflated to 50 mmHg above systolic pressure for 5 min to occlude brachial artery inflow and induce a flow stimulus. Vasodilation was evaluated at end-diastole. A high-resolution ultrasound system equipped with a 7.5-MHz linear transducer (Samsung HS30, Samsung, Seoul, Republic of Korea) and electronic caliper was used for FMD measurements.

### 2.6. Statistical Analysis

- Passive smoke exposure group: Continuous variables were expressed as mean ± standard deviation; categorical variables as percentages. Normality was assessed with the Kolmogorov–Smirnov test. Between-group differences were analyzed using chi-square for categorical variables and ANOVA with Bonferroni correction for normally distributed variables. Bivariate correlations were evaluated by Spearman’s test. Variables with *p* < 0.10 were included in multivariate linear regression (stepwise procedure). Variables with *p* < 0.10 were included in multivariate linear regression (stepwise procedure). Multivariate linear regression models, adjusted for age, sex, BMI, mean arterial pressure and cotinine were used to compare non-smokers vs. conventional smokers, and non-smokers vs. HTP users. Statistical significance was set at *p* < 0.05. Analyses were performed using SPSS v25.0 (IBM, Armonk, NY, USA) and GraphPad Prism 7 (GraphPad Software, San Diego, CA, USA). Sample size was calculated based on a two-sided Student’s t-test: a difference (δ) of 2.5% in FMD between exposed and unexposed children, standard deviation of 2.5%, α = 0.05, and power (1 − β) = 0.95, yielding 26 patients per group.
- Active smokers group: Continuous variables were reported as median (IQR), categorical as counts (%). Group comparisons were performed using Kruskal–Wallis tests and Wilcoxon–Mann–Whitney tests. Correlations were analyzed by Spearman’s test and visualized with clustered heatmaps. Multivariate linear regression models, adjusted for age, sex, BMI, mean arterial pressure, total cholesterol, and cotinine, were used to compare non-smokers vs. conventional smokers, and non-smokers vs. HTP users. Comparisons between conventional smokers and HTP users were further adjusted for smoking duration and cigarettes/day. A two-tailed p < 0.05 was considered statistically significant. Analyses were conducted with SPSS v18.0 (SPSS, Chicago, IL, USA), Stata 13 (StataCorp, College Station, TX, USA), and R 3.5.3 (R Foundation for Statistical Computing, Vienna, Austria).

## 3. Results

### 3.1. Passive Smoking

In the first part of the study, we analyzed children exposed to passive smoke from traditional tobacco cigarettes, heated tobacco products, and a control group. The clinical characteristics of the three groups are summarized in Table 1. As previously reported, there were no significant differences among the groups in terms of age, fasting blood glucose, systolic and diastolic blood pressure, or body mass index (BMI) (Table 1). As expected, children exposed to passive smoking had significantly higher serum cotinine levels compared to the control group (Table 1). No significant difference in serum cotinine levels was observed between the traditional tobacco (TT) and HTPs groups [12].

**Figure 1:**
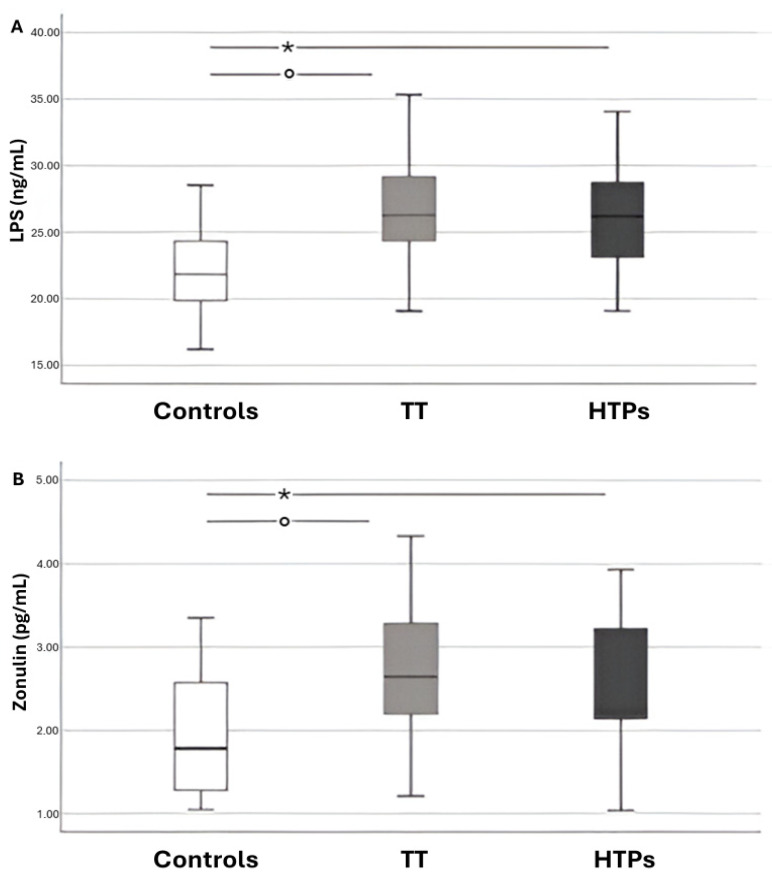
Box–and–whisker plots showing data from children divided into three groups: controls, those exposed to traditional tobacco cigarette smoke (TT), and those exposed to heated tobacco product emissions (HTPs). Panel **A** displays circulating lipopolysaccharide (LPS) levels, while Panel **B** shows circulating zonulin levels. The boxes represent the interquartile range (IQR; 25th–75th percentile), with the horizontal line indicating the median. Whiskers extend to values from the box to the minimum and maximum values. Panel (**A**): * *p* < 0.001; ° *p* < 0.001. Panel (**B**): * *p* = 0.006; ° *p* = 0.003.

Serum levels of LPS and zonulin were significantly elevated in children exposed to passive smoke from both traditional and heated tobacco sources (Figure 1). No statistically significant differences were observed between children exposed to heated tobacco product (HTP) emissions and those exposed to conventional cigarette smoke.

Simple linear regression analysis showed that serum LPS levels were positively correlated with zonulin (Rs = 0.580, *p* < 0.001), sCD40L (Rs = 0.224, *p* = 0.04), p-selectin (Rs = 0.286, *p* = 0.01) and cotinine (Rs = 0.550, *p* < 0.001), and negatively correlated with HBA (Rs = −0.325, *p* < 0.001).

Multivariable linear regression analysis identified cotinine (standardized coefficient β = 0.343; SE = 0.029; *p* = 0.005) and zonulin (standardized coefficient β = 0.441; SE = 0.452; *p* < 0.001) as the only independent predictors significantly associated with LPS levels (R^2^ = 0.20) (Table 2).

### 3.2. Active Smokers

In the second part of the study, we investigated the effects of smoking on low-grade endotoxemia in a population of cigarette smokers, including users of heated tobacco products, conventional tobacco, and healthy controls [11].

The clinical characteristics of this population are reported in Table 1.

**Figure 2:**
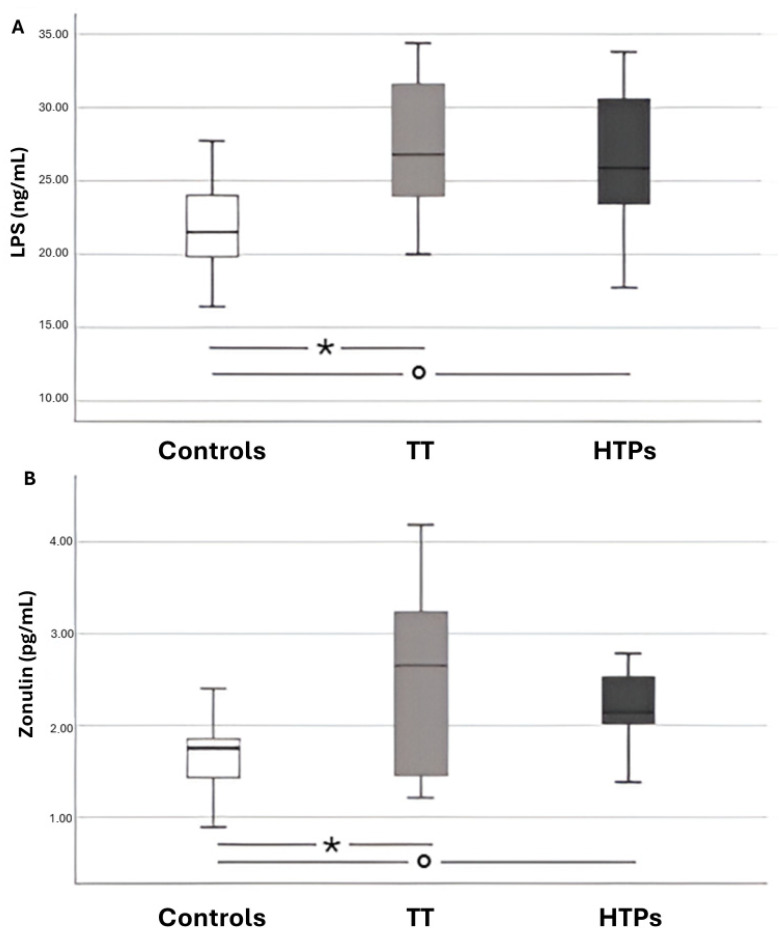
Box-and-whisker plots illustrating data from adults across three groups: healthy controls, chronic smokers of conventional tobacco cigarettes (TT), and users of heated tobacco products (HTPs). Panel **A** shows circulating levels of LPS, while Panel **B** reports circulating levels of zonulin. Panel (**A**): * *p* < 0.001; ° *p* = 0.001. Panel (**B**): * *p* = 0.002; ° *p* < 0.001.

No statistically significant differences were observed between the two types of active smokers (traditional tobacco vs. heated tobacco) in terms of LPS and zonulin levels. However, significant differences were found when comparing both smoking groups to the control subjects (Figure 2).

A bivariate analysis revealed a linear correlation between LPS and the following variables: zonulin (Rs = 0.581, *p* < 0.001), H_2_O_2_ (Rs = 0.398, *p* = 0.002), NOX2 (Rs = 0.494, *p* < 0.001), NO (Rs = −0.520, *p* < 0.001), sCD40L (Rs = 0.286, *p* = 0.02), P-selectin (Rs = 0.437, *p* < 0.001), and cotinine (Rs = 0.485, *p* < 0.001).

A multiple linear regression analysis identified zonulin (standardized coefficient β = 0.477; SE = 0.751; *p* < 0.001) and nitric oxide (standardized coefficient β = −0.307; SE = 0.033; *p* = 0.007) as independently associated with serum LPS levels (Table 3).

## 4. Discussion

This study demonstrates that exposure to both passive and chronic smoking, from either HTPs or conventional cigarettes, increases low-grade endotoxemia. To our knowledge, circulating LPS levels have not previously been investigated in either conventional cigarette smokers or HTP users, making our results particularly novel.

The effects of conventional cigarette smoking on the gastrointestinal tract are well established. Cigarette smoke contains thousands of chemicals, including reactive aldehydes, hydrocarbons, and free radicals, which exert toxic effects not only on the lungs but also on the gut. Previous studies have shown that smoking alters the composition of the gut microbiota, reduces microbial diversity, and increases the relative abundance of potentially pathogenic species [18]. At the same time, smoking promotes systemic and local inflammation and oxidative stress. These changes may explain the elevated levels of circulating LPS observed in smokers in our study, since LPS originates from the outer membrane of Gram-negative bacteria and enters the circulation when barrier integrity is compromised [19].

Animal models provide strong mechanistic support. Chronic smoke exposure has been shown to damage intestinal villi, reduce tight junction proteins such as claudins and occludin, and promote bacterial translocation [20,21]. These structural and functional changes collectively lead to “leaky gut,” which facilitates the entry of LPS into the bloodstream [20,21]. Other studies demonstrated increased gut permeability and serum LPS in mice exposed to cigarette smoke, confirming a causal link between smoking and endotoxemia. In addition to direct mucosal damage, smoke-induced hypoxia may represent another mechanism. Cigarette smoke reduces oxygen availability, and hypoxic conditions activate hypoxia-inducible factors (HIF-1α), which impair epithelial renewal, promote vascular dysfunction, and exacerbate barrier leakiness [22,23,24,25]. Furthermore, both mainstream and sidestream smoke contain measurable amounts of LPS, which may provide a direct exogenous source of endotoxin [26].

A novel aspect of our study is the evaluation of HTPs. These devices are marketed as reduced-risk alternatives to conventional cigarettes because they heat rather than burn tobacco. However, our findings challenge this assumption, showing that HTP exposure is also associated with increased circulating LPS and zonulin levels. The observation that passive exposure to HTP emissions results in similar changes emphasizes the potential health risks for non-users.

The mechanism underlying these findings may involve gut microbiota dysbiosis. Tian et al. demonstrated that 90-day HTP exposure in mice significantly reduced Lactobacillus abundance, a genus known to support epithelial integrity, produce anti-inflammatory metabolites, and compete against pathogens [27]. Reduced Lactobacilli could predispose to increased LPS release and absorption [27,28,29,30]. Our data, showing elevated LPS and zonulin in HTP users, are consistent with this interpretation. Interestingly, industry-sponsored studies have reported opposite results, with increased Lactobacilli following the switch from cigarettes to HTPs [31]. Discrepancies may reflect differences in experimental design, exposure duration, or conflicts of interest. Independent replication in human cohorts is urgently needed.

Zonulin plays a central role in the regulation of intestinal permeability by reversibly disassembling tight junctions [32]. Elevated serum zonulin is considered an indirect biomarker of enhanced permeability [32]. In our study, zonulin levels were increased in both conventional and HTP smokers, as well as in passively exposed individuals, strongly supporting the notion of barrier dysfunction. Importantly, the correlation between zonulin and LPS suggests that smoke-induced permeability changes may directly mediate systemic endotoxemia. However, as zonulin is not a perfect marker, and LPS could potentially derive from extra-intestinal sites such as the oral cavity or the lungs, further mechanistic investigations are required.

**Figure 3:**
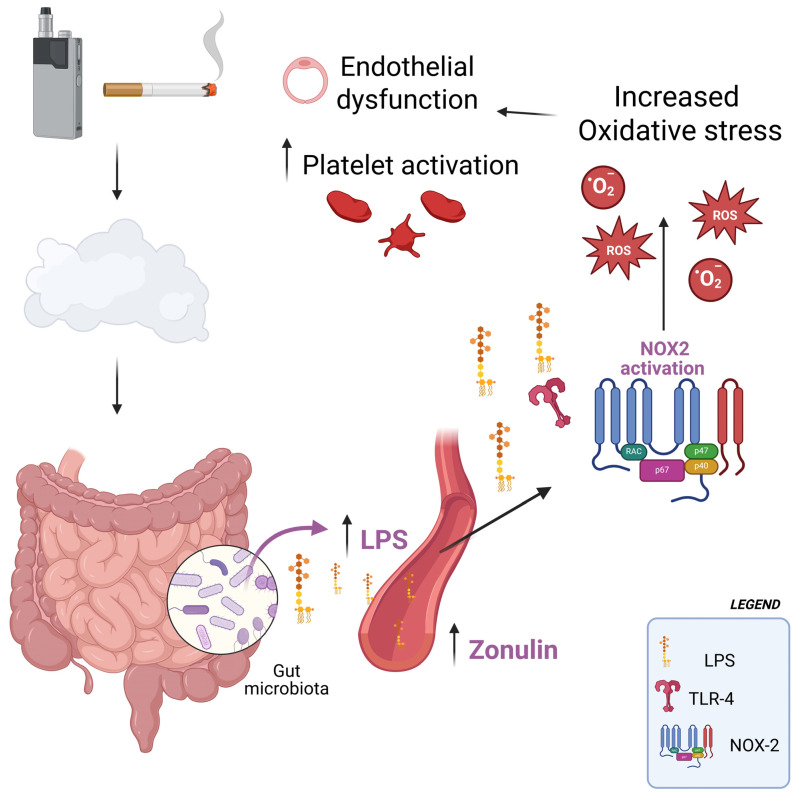
Following exposure to first-, second-, or third-hand smoke—whether derived from heated tobacco products or from conventional combustible cigarettes—there is an increase in circulating levels of lipopolysaccharides (LPS), most likely as a consequence of enhanced intestinal epithelial permeability. Elevated concentrations of even low-grade LPS promote the activation of the enzyme NADPH oxidase 2 (NOX2), which in turn drives excessive production of reactive oxygen species (ROS). This oxidative imbalance contributes to endothelial dysfunction and enhances platelet activation, two interrelated processes that are widely recognized as pivotal mechanisms in the development and progression of cardiovascular risk.

In our previous studies [11,12], we demonstrated that exposure to both conventional cigarette smoke and heated tobacco products induces oxidative stress, endothelial dysfunction, and platelet activation in exposed subjects. In the current study, the pathophysiological implications of elevated LPS are significant. LPS activates TLR4 on immune and endothelial cells, initiating an inflammatory cascade mediated by NF-κB [33]. In our study, circulating LPS correlated with NOX2 activation, hydrogen peroxide levels, reduced nitric oxide bioavailability, sCD40L and p-selectin. This suggests a mechanistic link in which smoke-induced endotoxemia activates NOX2, thereby amplifying oxidative stress, impairing endothelial function, and promoting platelet activation, as evidenced by the simple and multiple linear correlations observed in this study. Such processes are central in the pathogenesis of atherosclerosis and thrombosis, providing a plausible explanation for the increased cardiovascular risk observed in smokers (Figure 3). An additional potential influence on this pathological process may have been the exposure of study participants to nicotine. Previous research has shown that nicotine induces dysbiosis, which could, in turn, contribute to elevated circulating levels of LPS [34,35]. Although HTPs do not involve combustion as TTs do, they may still exert comparable deleterious effects on intestinal integrity, potentially leading to similar levels of LPS release into the bloodstream [36]. This appears plausible considering that both TTs and HTPs contain comparable nicotine concentrations, and that individuals who switch from TTs to HTPs often increase their overall consumption [37]. Furthermore, it should be noted that HTPs reach temperatures high enough to enable nicotine absorption through pyrolysis processes.

Our study has limitations. The sample size was relatively small, and larger studies are needed to validate these results. The mechanistic pathways of LPS translocation were not directly examined, and we cannot exclude alternative sources. We also did not assess gut microbiota composition, which would have provided direct evidence for dysbiosis. Nonetheless, the consistent increase in both LPS and zonulin, and their correlation, supports the hypothesis of impaired gut barrier function; however, Zonulin serves as an indirect marker, and we cannot rule out other potential sources of serum LPS. Another limitation of the study is the lack of data on the duration of conventional cigarette use prior to switching to HTPs. The participants’ long-term history of traditional cigarette use before transitioning to heated tobacco products may have confounded or influenced the results observed in the HTPs user group. Further studies involving smokers who have exclusively used HTPs are needed to assess this potential influence.

The clinical implications are considerable. Low-grade endotoxemia has been linked to metabolic syndrome, insulin resistance and cardiovascular disease. The fact that both conventional and HTPs exposure, including passive exposure, increase endotoxemia underscores the importance of public health interventions. While dietary strategies such as probiotics [38], prebiotics or antioxidants may help reduce endotoxemia [39], smoking cessation and avoidance of passive exposure remain the most effective strategies to prevent the downstream consequences of LPS elevation.

Future research should address several points: (i) longitudinal studies to determine whether LPS and zonulin levels normalize after smoking cessation or switching from HTPs to abstinence; (ii) microbiome sequencing to identify specific bacterial taxonomy involved; (iii) mechanistic studies linking LPS-TLR4 signaling to NOX2 activation and cardiovascular outcomes; and (iv) interventional trials testing whether microbiota-targeted therapies can mitigate smoke-induced endotoxemia.

In conclusion, this study demonstrates that both conventional cigarettes and HTPs, including passive exposure, are associated with increased low-grade endotoxemia and elevated zonulin levels. These alterations likely indicate impaired gut barrier integrity and may contribute to NOX2-mediated oxidative stress, endothelial dysfunction, and heightened cardiovascular risk. Therefore, heated tobacco products, much like traditional cigarettes, may exert a detrimental effect on low-grade endotoxemia and related pathophysiological processes.

## Figures and Tables

**Table 1:** Characteristics of the children and adult cohorts.

**Children**			
**Variable**	**Controls (N = 26)**	**TT (N = 26)**	**HTPs (N = 26)**
Age	9 ± 3	9 ± 3	10 ± 3
Female Gender	19 (73%)	16 (62%)	17 (65%)
FMD (%)	7.93 ± 2.30	5.78 ± 2.92	5.51 ± 3.00
NO (μM)	60.69 ± 11.44	48.12 ± 11.15	49.92 ± 9.01
sNox2-dp (pg/mL)	17.65 ± 7.92	25.96 ± 5.26	24.87 ± 7.64
H_2_O_2_ (μM)	23.19 ± 5.41	32.35 ± 7.61	29.04 ± 6.13
sCD40L (ng/mL)	1.83 ± 0.48	2.78 ± 0.81	2.44 ± 0.84
sP-selectin (ng/mL)	5.10 ± 1.74	6.77 ± 1.92	6.33 ± 1.20
Platelet aggregation (%)	25.60 ± 3.78	39.60 ± 5.90	37.00 ± 7.18
Cotinine (ng/mL)	1.45 ± 1.25	36.59 ± 9.42	33.46 ± 6.58
LPS (pg/mL)	21.95 ± 3.01	25.89 ± 3.95	26.88 ± 4.31
Zonulin (ng/mL)	1.94 ± 0.77	2.55 ± 0.86	2.71 ± 0.90
8-iso-PGF2α (pg/mL)	142.50 ± 20.89	176.43 ± 43.75	178.5 ± 36.26
HBA (%)	53.37 ± 7.68	40.95 ± 6.37	45.11 ± 5.73
**Adults**			
**Variable**	**Controls (N = 20)**	**TT (N = 20)**	**HTPs (N = 20)**
Age	28 (23–33)	27 (24– 30)	33 (28–44)
Female Gender	11 (55%)	10 (50%)	12 (60%)
FMD (%)	7.1 (2.8–11.5)	1.6 (0–3.9)	3.3 (2.4–6.0)
NO (μM)	41 (38–49)	10 (9–13)	10 (8–13)
sNox2-dp (pg/mL)	19 (15–23)	46 (41–50)	40 (34–41)
H_2_O_2_ (μM)	8.8 (7.2–11.9)	33.5 (19.5–52.7)	26.7 (21.9–33.8)
sCD40L (ng/mL)	1.6 (1.1–2.1)	3.2 (2.5–4.4)	3.0 (2.5–3.3)
sP-selectin (ng/mL)	3.0 (2.0–3.9)	9.2 (6.7–12.0)	8.1 (5.5–9.2)
Platelet aggregation (%)	62 (58–70)	80 (77–80)	76 (70–80)
Cotinine (ng/mL)	2 (2–3)	139 (130–148)	137 (103–163)
LPS (pg/mL)	21.68 ± 4.82	26.62 ± 4.58	27.43 ± 4.31
Zonulin (ng/mL)	1.68 ± 0.38	2.28 ± 0.53	2.55 ± 0.92

**Table 2:** Multiple linear regression for the dependent variable LPS in children. (β = regression coefficient; SE = standard error).

	β	SE	*p*
Zonulin (ng/mL)	0.441	0.452	<0.001
sCD40L (ng/mL)	0.042	0.504	0.665
sP-Selectin (ng/mL)	0.017	0.262	0.861
HBA (%)	−0.027	0.054	0.798
Cotinin (ng/mL)	0.343	0.029	0.005

**Table 3:** Multiple linear regression for the dependent variable LPS in adults. (β = regression coefficient; SE = standard error).

	β	SE	*p*
Zonulin (ng/mL)	0.477	0.751	<0.001
NO (µM)	−0.307	0.033	0.007

## Data Availability

The original contributions presented in this study are included in the article Further inquiries can be directed to the corresponding author (Request to: Prof. Lorenzo Loffredo; e-mail: lorenzo.loffredo@uniroma1.it).
